# Screening on Perpetration and Victimization of Intimate Partner Violence (IPV): Two Studies on the Validity of an IPV Screening Instrument in Patients in Substance Abuse Treatment

**DOI:** 10.1371/journal.pone.0063681

**Published:** 2013-05-16

**Authors:** Fleur L. Kraanen, Ellen Vedel, Agnes Scholing, Paul M. G. Emmelkamp

**Affiliations:** 1 Department of Clinical Psychology, University of Amsterdam, Amsterdam, The Netherlands; 2 Forensic Outpatient Clinic De Waag, Amsterdam, The Netherlands; 3 Jellinek Substance Abuse Treatment Centre, Amsterdam, The Netherlands; 4 Department of Psychology, King Abdulaziz University, Jeddah, Saudi Arabia; Catholic University of Sacred Heart of Rome, Italy

## Abstract

**Background:**

About 50% of patients in substance abuse treatment with a partner perpetrated and/or experienced intimate partner violence in the past year. To date, there are no screeners to identify both perpetrators and victims of partner intimate violence in a substance abusing population. We developed a 4 item screening instrument for this purpose, the Jellinek Inventory for assessing Partner Violence (J-IPV). Important strengths of the J-IPV are that it takes only 2 minutes to administer and is easy to use and to score.

**Methods:**

To investigate the validity of the J-IPV, two independent studies were conducted including 98 and 99 participants, respectively. Aim of the second study was to cross-validate findings from the first study. Psychometric properties of the J-IPV were determined by calculating sensitivity, specificity, positive and negative predictive value, and positive and negative likelihood ratio’s by comparing J-IPV outcomes to outcomes on the Revised Conflict Tactics Scales (‘gold standard’). Also, receiver operator characteristics (ROC)-curves were determined to weight sensitivity and specificity as a result of different J-IPV cutoffs, and the area under the curve (AUC) was calculated.

**Results:**

Results of the first study demonstrated that the J-IPV possesses good psychometric properties to detect perpetrators and victims of *any* as well as *severe* intimate partner violence. Results from the second study replicated findings from the first study.

**Conclusions:**

We recommend administering the J-IPV to patients entering substance abuse treatment. If perpetrators and victims of partner violence are identified, action can be taken to stop IPV perpetration and arrange help for victims, for example by offering perpetrators treatment or by providing safety planning or advocacy interventions to victims.

## Introduction

Intimate partner violence (IPV) is a serious problem that comprises verbal, physical, and/or sexual violence against an (ex-)partner; the present study focuses on physical IPV. Consequences of physical IPV can be severe for victims and may result in injuries, chronic pain, depression, posttraumatic stress disorder, suicidality, and substance use disorders [1], [2]. In addition, witnessing IPV between parents may lead, for example, to anger, fear, posttraumatic stress disorder, depression, and conduct problems in children (e.g., [3]–[5]). Moreover, these children are at greater risk for IPV perpetration and/or victimization as an adult (e.g., [5]). IPV is also a prevalent problem. In the US, for example, about one fifth of the couples experienced physical IPV in the past year [6]; Dutch figures appear somewhat lower [7]. In addition, a large proportion of IPV perpetrators are diagnosed with substance use disorders. Substance abuse and dependence are highly prevalent among IPV perpetrators in domestic violence treatment (e.g., [8]–[10]), and approximately 35–60% of male and female patients in substance abuse treatment committed at least one act of *physical* IPV in the past year (e.g., [11]–[18]).

Because of the serious consequences of IPV and its high prevalence among patients in substance abuse treatment, it is important to assess IPV perpetration among these individuals. Ideally, IPV perpetrators should be identified at intake in order to prevent further assault of victims [19]. Worldwide, the Conflict Tactics Scales (CTS) [20] and Revised Conflict Tactics Scales (CTS2) [21] are the most used instruments to assess perpetration and victimization of IPV [22]. Reliability and validity of the CTS and CTS2 are moderate to high [20], [23]–[27]. However, these instruments consist of 38 and 78 items respectively and take considerable time for patients to complete (i.e., 12–15 minutes for the CTS2; [22]). These long questionnaires are often problematic to implement in an intake, which usually lasts only 45–60 minutes. A short version of the CTS2 (CTS2S; [28]) is available, but this instrument still comprises of 20 items (10 items addressing IPV perpetration, 10 items addressing IPV victimization). [28] claimed that completion only takes 3 minutes, but our clinical experience is that this is only true for patients who do not experience IPV and thus have little to report on the CTS2S. A systematic review pointed out that limited time was an important barrier to screening for IPV in health care settings [29], which implies that there is a clear need for a screener to detect IPV perpetration that takes little time to administer. To date, such a screener is lacking. Given that insufficient knowledge and training also withheld healthcare workers from screening for IPV [29], an IPV screener should have a simple response scale and scoring method. Further, because [29] reported healthcare workers’ personal discomfort as one of the barriers for screening for IPV, we assumed that the use of a standardized screener to address IPV would facilitate intakers to discuss this sensitive subject. Finally, we decided that an IPV screener should have the format of a structured interview, to facilitate its use during a (structured) intake.

On the basis of the criteria described above we developed the 4 items counting Jellinek Inventory for assessing Partner Violence (J-IPV), a screener that takes only 2 minutes to administer, and is simple to use and score. The first two questions of the J-IPV address *victimization* of IPV in the past year; the latter two questions address *perpetration* of IPV in the past year (see Appendix S1). The items addressing IPV victimization were included in order to bring about the subject of IPV in a non-offensive manner, as it is our clinical experience that it is easier for both men and women to report that their partner was offensive to them. Moreover, the fact that IPV can be reciprocal (both partners being perpetrator as well as victim) [30], [31] urges to ask about both IPV perpetration and victimization. Our first aim was to investigate the validity of the J-IPV in distinguishing patients in substance abuse treatment who did and who did not commit physical IPV in the past year by comparing outcomes on the J-IPV to outcomes on the CTS2 (‘gold standard’). Since the J-IPV also inquires about IPV *victimization*, and substance abuse is associated with IPV victimization as well (e.g., [13], [32]–[38]), it was also studied whether the J-IPV was able to discriminate between patients in substance abuse treatment who experienced *victimization* of IPV in the past year and those who did not. Although several tools to screen for IPV victimization were available, (e.g., the Hurt, Insult, Threaten, Scream (HITS) [39], the Women Abuse Screening Tool (WAST) [40], and the Partner Violence Screen (PVS) [41] (for reviews, see: [42], [43]), none of these have been validated in a substance abusing population.

To determine psychometric properties of the J-IPV for detecting 1) any IPV perpetration, 2) severe IPV perpetration, 3) any IPV victimization, and 4) severe IPV victimization, the following values were determined: 1) sensitivity (proportion of factual IPV perpetrators who indeed screen positive), 2) specificity (proportion of factual non-IPV perpetrators who indeed screen negative), 3) positive predictive value (PPV; proportion of all positive screening participants who actually committed IPV), and 4) negative predictive value (NPV; proportion of all negative screening participants screening who actually did not commit IPV) were computed. In addition, positive likelihood ratio (LR+; i.e., how much more likely a participant is to have perpetrated IPV after screening positive) and negative likelihood ratio (LR−; i.e., how much less likely a participant is to have committed IPV after screening negative) were calculated as suggested, for example, by [44] and [45]. Finally, receiver operator characteristics (ROC)-curves were determined and the area under the curve (AUC) was calculated to determine the probability that a randomly chosen IPV perpetrator obtained a higher score on the J-IPV than a randomly chosen non-IPV perpetrator.

There are no universal criteria to determine the minimum standards of a screening instrument, since it depends on the situation whether greater value is attached to sensitivity or specificity [46]. However, [47] suggested that clinicians and policy makers should select screening instruments that have both sensitivity and specificity of at least.80. In case of assessing IPV, we argued that high sensitivity (detecting all factual perpetrators and victims of IPV) and high NPV (the chance that after screening negative, IPV perpetration or victimization has indeed not taken place) are the most important qualities the J-IPV should possess. It is essential that if a patient screens negative on the J-IPV, it is safe to assume that indeed no IPV took place in the past year. Therefore, we hypothesized that, in order to be clinically useful, the J-IPV should possess sensitivity and NPV ≥.80. Since validity coefficients capitalize on random errors within a specific sample [48], classical test development requires that the performance of an instrument is confirmed in a cross-validation study [49]. For that reason, two methodological identical studies in two different settings were conducted in order to calculate the aforementioned psychometric properties; aim of the second study was to cross-validate findings from the first study. Because of these random sampling errors, it is expected that the second study performs less than the first study [48]. However, psychometric properties of the J-IPV should remain acceptable (i.e., sensitivity and NPV ≥.80).

## Materials and Methods

### Ethics Statement

The study was approved by the ethical committee of the University of Amsterdam (reference number 2010-kp-1350). Written informed consent was obtained from all participants.

### Participants

The two studies were conducted at two different locations of Jellinek, a large substance abuse treatment facility, in Amsterdam (study 1) and Hilversum (study 2), the Netherlands. Patients were included if they had an intake between February 28^th^ 2011 and April 22^nd^ 2011 in Amsterdam or between December 28^th^ 2011 and October 17^th^ 2012 in Hilversum. Patients were included if they 1) fulfilled DSM-IV-TR criteria for substance abuse or dependence (with the exclusion of nicotine dependence as the sole substance use disorder diagnosis), 2) had a partner for at least 3 months in the past year, 3) had sufficient knowledge of the Dutch language (i.e., could understand the J-IPV questions without additional explanation), and 4) were at least 18 years old. Patients were excluded in case of 1) severe withdrawal or intoxication symptoms during the intake, 2) severe mental illness (e.g., suicidal ideation or psychotic symptoms), and 3) severe cognitive disorders, such as Korsakoff’s syndrome or dementia.

### Instruments

#### Jellinek inventory for assessing partner violence (J-IPV)

The J-IPV is a 4 item-screening device that was developed to assess IPV perpetration in patients entering substance abuse treatment. FK formulated the items of the J-IPV and EV, AS, and PE independently reviewed the items; the final items were based on consensus. The questions are administered as a structured interview and questions are answered with ‘yes’ or ‘no’. Two native English speakers translated the J-IPV from Dutch into English. Translations were compared with one another and led to the final English version of the J-IPV (see Appendix S1).

#### Revised conflict tactics scales (CTS2)

The CTS2 [21] is the principal method to measure IPV among individuals in a lasting relationship [50] and was used as ‘gold standard’ to validate the J-IPV. The instrument consists of 39 item pairs addressing behavior that participant or partner may have exhibited when having a conflict with the other in the past year. Answers are scored on an 8 point scale, ranging from 0–7 (0 = never, 1 = once, 2 = twice, 3 = 3–5 times, 4 = 6–10 times, 5 = 11–20 times, 6 = more than 20 times, 7 = it has happened but not in the past year). The CTS2 contains items in five subscales, i.e., 1) verbal violence, 2) physical violence, 3) sexual violence, 4) negotiation, and 5) injuries resulting form a fight with the partner. An example of an item pair is: “I slapped my partner” and “My partner slapped me”. Since we intended the J-IPV to screen for *physical* IPV, we only used item pairs assessing physical violence (2×12 items). To make the transition to the physical violence items more gradual, firstly 3 items pairs addressing verbal violence were administered (‘I shouted or yelled at my partner’, ‘I insulted or swore at my partner’, ‘I threatened to hit or throw something at my partner’). Subsequently, the physical violence items of the CTS2 were administered in the same order in which they appear in the CTS2. The other CTS2 items were not used in this study. The physical violence items of the CTS2 were used to categorize participants as perpetrator and/or victim of any/severe physical IPV. Participants were rated as perpetrator of *any* IPV when answering ‘yes’ to at least one physical IPV perpetration item and were rated as victim of *any* IPV after at least one ‘yes’ to any of the physical IPV victimization items. Further, participants were classified as having perpetrated *severe* physical IPV or being victimized by severe physical IPV if at least one of the *severe* physical violence perpetration or victimization items was answered with “yes”. The adapted version of the CTS2 was administered as a structured interview, a method that was supported by [22]. Moreover, different studies found practically no differences between paper-and-pencil tests and interviews when assessing sensitive topics such as IPV (e.g., [51]–[54]).

#### Measurements in the addictions for triage and evaluation (MATE)

The MATE [55] was routinely administered during the intake to assess patient characteristics and to guide treatment allocation (stepped-care policy). In this study, data from the MATE were used to obtain demographics (age, nationality, education, having children under 18 years old, treatment intensity) and substance use disorder diagnoses according to DSM-IV/ICD-10.

### Procedure

During the intake, psychologists from the treatment staff who regularly conducted the intakes and were not involved in the factual studies administered the MATE. Subsequently, patients were informed about the study. If they agreed to participate, informed consent was obtained. Hereafter, the J-IPV and CTS2 were administered successively as structured interview. Since the aim of the study was to investigate whether the J-IPV predicted the outcome on the CTS2, we did not counterbalance administration of the J-IPV and CTS2. To help participants remember the 7-point rating scale of the CTS2, they were given a form with response categories written out. The first study was conducted at Jellinek Amsterdam; the second study at Jellinek Hilversum in order to cross-validate findings from the first study.

### Data Analyses

Demographics of participants of the 2 studies were compared using chi-square tests for categorical variables (gender, nationality, living together, relationship intact, having children, treatment intensity and primary SUD diagnosis); an independent samples t-test for normally distributed continuous variables (age); and a Mann-Whitney test for nonnormally distributed continuous variables (relationship length). To determine psychometric properties of the J-IPV, sensitivity, specificity, PPV, NPV, LR+, LR−, and AUC were calculated for the J-IPV compared to the CTS2 as ‘gold standard’ (see Table 1 for a comparison of all indicators).

**Table 1:** Formulas used to calculate sensitivity, specificity, PPV, NPV, LR+, and LR−.

	IPV perpetrator/victim	Non-IPV perpetrator/victim		
Screen positive perpetration/victimization	A (true positives)	B (false positives)		PPV = A/A+B
Screen negative perpetration/victimization	C (false negatives)	D (true negatives)		NPV = D/D+C
	Sensitivity = A/A+C	Specificity = D/B+D		
LR+ = sensitivity/(1– specificity)			LR− = (1– sensitivity)/specificity	

IPV = intimate partner violence; PPV = positive predictive value; NPV = negative predictive value; LR+ = positive likelihood ratio; LR− = negative likelihood ratio.

Prior to the start of this study, the J-IPV had been used in clinical practice for 20 months. Clinical observations taught that some patients who had committed physical IPV but saw themselves primarily as victim (and not perpetrator), did answer positively to one or both victimization items, but not to any of the perpetrator items. This demonstrates that people tend to underreport IPV perpetration (e.g., [56]) but also shows that indeed IPV is often reciprocal [33]; [34]. Therefore, psychometric properties of the J-IPV to detect any/severe IPV perpetration were determined when 1) *all* J-IPV items were used, 2) only both J-IPV *perpetration* items were used (items 3 and 4) and 3) the *separate* items addressing IPV perpetration (items 3 and 4) were used. Psychometric properties of the J-IPV to detect any/severe IPV victimization were determined when 1) *all* J-IPV items were used, 2) only both J-IPV *victimization* items were used (items 1 and 2) and 3) the *separate* items addressing IPV victimization (items 1 and 2) were used. After determining psychometric properties for these different options, the optimal cutoff or scoring method was selected to classify participants as 1) perpetrator of any IPV, 2) perpetrator of severe IPV, 3) victim of any IPV, and 4) victim of severe IPV. As mentioned in the introduction, we valued high sensitivity and NPV the most important psychometric properties the J-IPV should possess because it might be harmful to overlook perpetrators and victims of IPV. Therefore, the following steps were taken to decide on the optimal cutoff or scoring method. First, it was examined which cutoff or scoring method resulted in highest sensitivity and NPV. Then, it was observed whether this scoring method also yielded the highest AUC, and finally it was examined whether specificity and PPV were still acceptable.

## Results

### Participants

#### Study 1

**Figure 1:**
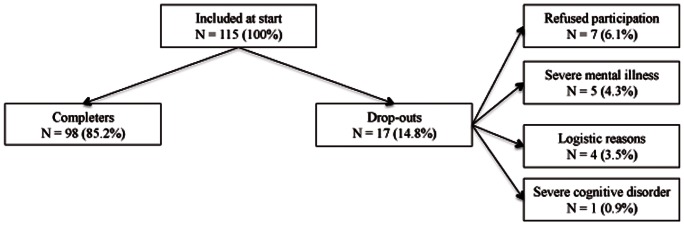
Overview of participants and drop-outs of study 1.

A total of 115 participants met inclusion criteria. Seventeen participants (14.8%) were excluded: 7 participants (6.1%) refused participation, 5 (4.3%) suffered from severe mental illness, 4 (3.5%) dropped-out because of logistic reasons, and 1 (0.9%) suffered from a severe cognitive disorder (see Figure 1). The final sample consisted of 98 participants (85.2%). Demographics and past year prevalence of any/severe IPV perpetration/victimization as determined with the CTS2 are displayed in Table 2.

**Table 2:** Demographic variables and prevalence of IPV as determined with the CTS2 of participants in study 1 and study 2.

	Study 1	Study 2	Total
	N = 98	N = 99	N = 197
	N (%)	N (%)	N (%)
Gender			
* Male*	69 (70.4)	66 (66.7)	135 (68.5)
* Female*	29 (29.6)	33 (33.3)	62 (31.5)
Age (M, SD)	42.6 (10.92)	40.69 (11.91)	41.64 (11.44)
Nationality			
* Dutch*	88 (89.8)	97 (98.0)	185 (94.9)
* Other European*	2 (2.0)	1 (1.0)	3 (1.5)
* Other outside Europe* *	8 (8.2)	1 (1.0)	9 (4.6)
Relationship length (year) (M, SD)*	8.31 (10.32)	10.56 (11.50)	9.48 (10.98)
Living together	54 (57.1)	66 (66.7)	120 (60.9)
Relationship intact at the time of the intake	80 (81.6)	79 (79.8)	159 (80.7)
Children <18 years old	41 (41.8)	47 (48.0)	88 (44.7)
Treatment intensity^3^			
* Outpatient*	89 (90.8)	87 (87.9)	176 (89.3)
* Inpatient*	9 (9.2)	12 (12.1)	21 (10.7)
Primary SUD diagnosis			
* Alcohol abuse or dependence*	67 (68.4)	68 (68.7)	135 (68.5)
* Cannabis abuse or dependence*	16 (16.3)	15 (15.2)	31 (15.7)
* Cocaine abuse or dependence*	11 (11.2)	12 (12.1)	23 (11.7)
* Other substance abuse or dependence*	4 (4.1)	4 (4.0)	8 (4.1)
Any past year IPV perpetration	41 (41.8)	38 (38.4)	79 (40.1)
Any past year IPV victimization	42 (42.9)	40 (40.4)	82 (41.6)
Severe past year IPV perpetration	18 (18.4)	15 (15.2)	33 (16.8)
Severe past year IPV victimization	18 (18.4)	15 (15.2)	33 (16.8)

CTS2 = Revised Conflict Tactics Scales; SUD = substance use disorder;

1 Positive screener: one or more items of the J-IPV answered with ‘yes’;

2 Negative screener: none of the J-IPV items answered with ‘yes’;

3 after the intake, patients were assigned to either inpatient or outpatient treatment;

* = p<.05.

On the basis of the CTS2, participants were classified as 1) no perpetrator, no victim, 2) perpetrator only, 3) victim only, and 4) both perpetrator and victim of physical IPV (see Table 3). About half of the participants committed and/or experienced IPV in the past year. In accordance with previous research [30], [31], in most cases, IPV was reciprocal (69.4% of the sample in which IPV had taken place; 34.7% of the total sample).

**Table 3:** Distribution of participants who perpetrated IPV, experienced IPV victimization, who were both perpetrator and victim of IPV and who were nor perpetrator, nor victim in the past year of study 1 and study 2.

	Study 1			Study 2		
	IPV perpetrator based on CTS2			IPV perpetrator based on CTS2		
IPV victim based on CTS2	Yes	No	Total	Yes	No	Total
	N (%)	N (%)	N (%)	N (%)	N (%)	N (%)
Yes	34 (34.7)	8 (8.2)	42 (42.9)	33 (33.3)	7 (7.1)	40 (40.4)
No	7 (7.1)	49 (50.0)	56 (57.1)	5 (5.1)	54 (54.5)	59 (59.6)
Total	41 (41.8)	57 (58.2)	98 (100.0)	38 (38.4)	61 (61.6)	99 (100.0)

IPV = intimate partner violence; CTS2 = Revised Conflict Tactics Scales.

#### Study 2

**Figure 2:**
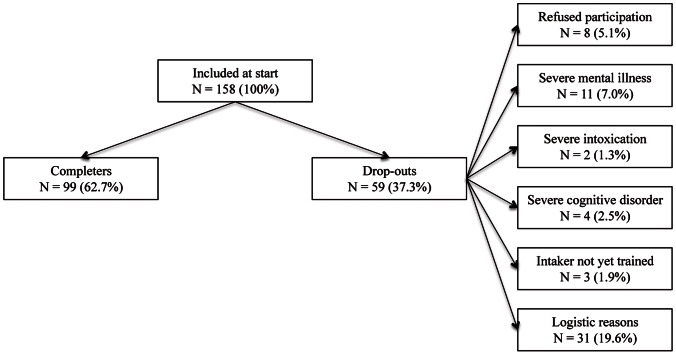
Overview of participants and drop-outs of study 2.

A total of 158 participants met inclusion criteria. Fifty-nine participants (37.3%) were excluded: 8 participants (5.1%) refused participation, 11 (7.0%) suffered from severe mental problems, 4 (2.5%) suffered from a severe cognitive disorder; 2 (1.3%) were severely intoxicated, 3 (1.9%) dropped-out because the intaker was not yet trained in administering the J-IPV and CTS2, and 31 (19.6%) dropped-out because of logistic reasons (see Figure 2). The final sample consisted of 99 participants (62.7%). Demographics and past year prevalence rates of any/severe IPV perpetration/victimization as determined with the CTS2 are displayed in Table 2 as well.

In addition, as in study 1, participants were classified as (non) perpetrator and/or victim of IPV (see Table 3). Again, in several cases IPV was reciprocal (73.7% of the sample in which IPV took place; 33.3% of the total sample). In total, 38.4% of participants of study 2 committed physical IPV in the past year and 40.4% of the participants experienced past year IPV.

#### Comparison of demographics and prevalence of IPV between study 1 and study 2

Demographics and IPV prevalence rates of participants of studies 1 and 2 were compared (see Table 2). Participants of studies 1 and 2 differed significantly regarding nationality (X^2^ (2) = 6.21; p<.05). Standardized residuals indicated that more participants of study 1 had a non-European nationality than participants in study 2. In addition, participants of study 1 and 2 differed significantly form one another regarding relationship length (F (186) = 4.52; p<.05); participants of study 2 had significantly longer relationships than participants of study 1. Participants of study 1 and 2 did not differ significantly regarding gender, age, living together, relationship at the time of the intake, children under 18, treatment intensity, and primary substance use disorder diagnosis. In addition, there were no differences between the 2 studies regarding prevalence rates of any/severe past year IPV perpetration/victimization. Also, there were no differences in rates of reciprocality of IPV (see Table 3).

### Effectiveness of the J-IPV for the Assessment of any IPV Perpetration

First, psychometric properties of the J-IPV for the assessment of *any* physical IPV perpetration were determined using CTS2 perpetration items as ‘gold standard’ (see Table 4) and were determined as follows: 1) diagnostic accuracy of *all* J-IPV items in identifying IPV perpetration as determined with the CTS2 was tested for different cutoff values, 2) psychometric properties of both J-IPV *perpetration* items (items 3 and 4) were calculated, and 3), diagnostic efficiency was calculated for items 3 and 4, separately (see Table 4). In study 1, a cutoff of 1 (a positive answer to *any* of the J-IPV items) resulted in highest sensitivity (.80) and NPV (.85) (the indicators that were decided to be most important to the J-IPV). Also, the largest AUC (.80) was obtained when all 4 J-IPV items were used. In addition, specificity (.80) and PPV (.75) were still acceptable for this option, as well as LR+ (4.17), and an LR− (.24). For study 2, similar results were found (sensitivity = .84; NPV = .89; AUC = .86; specificity = 82; PPV = .74; LR+ = , 4.67; LR− = .19) (see Table 4). For illustrative purposes, ROC-curves are displayed in Charts S1.

**Table 4:** Sensitivity, specificity, PPV, NPV, LR+, LR−, and AUC of the J-IPV as screener for IPV perpetration as compared with the CTS2 of study 1 and study 2.

	Any physical IPV perpetration														
	Study 1								Study 2						
	Cut-off	Sens.	Spec.	PPV	NPV	LR+	LR−	AUC (95% CI)	Sens.	Spec.	PPV	NPV	LR+	LR−	AUC (95% CI)
J-IPV	**1**	**.80**	**.80**	**.75**	**.85**	**4.17**	**.24**	**.86 (.78–.94)**	**.84**	**.82**	**.74**	**.89**	**4.67**	**.19**	**.86 (.78–.94)**
	2	.71	.95	.91	.82	14.2	.31	.86 (.78–.94)	.71	.90	.82	.83	7.22	.32	.86 (.78–.94)
Combination items 3 and 4	1	.71	.91	.85	.81	8.06	.32	.83 (.74–.92)	.68	.89	.79	.82	5.96	.36	.80 (.70–.90)
Individual items															
Item 3	–	.61	.93	.86	.77	8.71	.42	.77 (.67–.87)	.61	.89	.77	.78	5.27	.45	.75 (.64–.85)
Item 4	–	.56	.98	.96	.76	31.98	.45	.77 (.67–.87)	.50	.98	.95	.76	30.50	.51	.74 (.63–.85)
	**Severe physical IPV perpetration**														
J-IPV	1	1.00	.68	.41	1.00	3.08	0	.92 (.87–.98)	1.00	.67	.65	1.00	3.00	.00	.91 (.85–.97)
	**2**	**1.00**	**.83**	**.56**	**1.00**	**5.88**	**0**	**.92 (.87–.98)**	**1.00**	**.79**	**.45**	**1.00**	**4.67**	**.00**	**.91 (.85–.97)**
	3	.55	.89	.53	.90	5.00	.51	.92 (.87–.98)	.60	.93	.53	.91	8.20	.43	.91 (.85–.97)
Combination items 3 and 4	1	.94	.79	.50	.98	4.44	.07	.94 (.87–1.00)	1.00	.79	.45	1.00	4.67	.00	.94 (.89–.98)
Individual items															
Item 3	–	.89	.84	.55	.97	5.56	.13	.86 (.77–.96)	.94	.80	.47	.99	4.57	.08	.87 (.78–.96)
Item 4	–	.94	.91	.71	.99	10.79	.06	.93 (.86–1.00)	.80	.90	.60	.96	8.40	.22	.85 (.73–.98)

IPV = intimate partner violence; J-IPV = Jellinek Inventory for assessing Partner Violence; sens. = sensitivity; spec. = specificity; PPV = positive predictive value; NPV = negative predictive value; LR+ = positive likelihood ratio; LR− = negative likelihood ratio; AUC = area under the curve; ROC = receiver operator characteristics; CI = confidence interval.

### Effectiveness of the J-IPV for the Assessment of Severe IPV Perpetration

To detect severe IPV perpetration, using a cutoff of 2 resulted in optimal psychometric properties in study 1 (sensitivity = 1.00; NPV = 1.00; AUC = .92; specificity = .83; PPV = .56; LR+ = 5.88; LR− = .00, see Table 4). An alternative is to look only at item 4 when screening for severe IPV perpetration, which resulted in comparable psychometric properties (sensitivity = .94; NPV = .99; AUC = .93; specificity = .91; PPV = .71; LR+ = 10.79; LR− = .06). However, we prefer using a cutoff of 2 to identify perpetrators of severe IPV, because this resulted in less false negatives (scoring negative on the J-IPV while indeed someone has committed severe IPV). Even though the AUC of this option was not the largest, differences between AUC’s were only marginal. In study 2, using a cutoff of 2 resulted in optimal psychometric properties as well (sensitivity = 1.00; NPV = 1.00; AUC = .91; specificity = .79; PPV = .45; LR+ = 6.76; LR− = .00, see Table 4). However, PPV is somewhat low, given that only 45% participants who answered at least 2 J-IPV items with ‘yes’ indeed committed severe IPV. When a positive answer to J-IPV item 4 was used to determine severe IPV perpetration, values of psychometric properties obtained in study 2 were somewhat lower than values obtained in study 1 (sensitivity = .80; NPV = .96; AUC = .85; specificity = .90; PPV = .60; LR+ = 8.40; LR− = .22). ROC-curves are displayed in Charts S1.

### Effectiveness of the J-IPV for the Assessment of any IPV Victimization

Psychometric properties for detecting any physical IPV victimization using CTS2 victimization items as ‘gold standard’ are displayed in Table 5. Psychometric properties of the J-IPV were determined as follows: 1) diagnostic accuracy of *all* J-IPV items in identifying IPV victimization as determined with the CTS2 was tested for different cutoff values, 2) psychometric properties of both J-IPV *victimization* items (items 1 and 2) were calculated, and 3) diagnostic efficiency fore detecting IPV victimization was calculated for the separate victimization items (1 or 2). A cutoff of 1 resulted in the most favorable psychometric properties (sensitivity = .83; NPV = .87; AUC = .85; specificity = .84; PPV = .80; LR+ = 5.19; LR− = .20). Similar results were obtained in study 2 (sensitivity = .80; NPV = .86; AUC = .84; specificity = .81; PPV = .74; LR+ = 4.29; LR− = .25). ROC-curves are displayed in Charts S1.

**Table 5:** Sensitivity, specificity, PPV, NPV, LR+, LR−, and AUC of the J-IPV as screener for IPV victimization as compared with the CTS2 of study 1 and study 2.

	Any physical IPV victimization														
	Study 1								Study 2						
	Cut-off	Sens.	Spec.	PPV	NPV	LR+	LR−	AUC (95% CI)	Sens.	Spec.	PPV	NPV	LR+	LR−	AUC (95% CI)
J-IPV	**1**	**.83**	**.84**	**.80**	**.87**	**5.19**	**.20**	**.85 (.76–.93)**	**.80**	**.81**	**.74**	**.86**	**4.29**	**.25**	**.84 (.76–.93)**
	2	.60	.88	.78	.75	5.00	.45	.85 (.76–.93)	.68	.90	.82	.80	6.64	.36	.84 (.76–.93)
Combination items1 and 2	1	.69	.94	.91	.80	12.89	.33	.82 (.73–.91)	.68	.92	.84	.81	7.97	.26	.80 (.71–.90)
Individual items															
Item 1	–	.60	.95	.89	.76	12.00	.42	.77 (.67–.87)	.58	.92	.82	.76	6.79	.46	.75 (.64–.85)
Item 2	–	.52	.96	.92	.73	14.67	.49	.75 (.64–.85)	.60	.97	.92	.78	17.70	.41	.78 (.68–.89)
	**Severe physical IPV victimization**														
J-IPV	1	1.00	.68	.41	1.00	3.08	0	.89 (.83–.96)	1.00	.67	.35	1.00	3.00	.00	.94 (.90–.99)
	2	.89	.80	.50	.97	4.45	.14	.89 (.83–.96)	1.00	.79	.45	1.00	4.67	.00	.94 (.90–.99)
	3	.61	.90	.58	.91	6.10	.43	.89 (.83–.96)	.73	.91	.65	.95	7.88	.29	.94 (.90–.99)
Combination items 1 and 2	1	1.00	.83	.56	1.00	5.71	0	.93 (.88–.98)	1.00	.80	.47	1.00	4.94	.00	.93 (.89–.98)
	2	.72	.91	.65	.94	8.00	.20	.93 (.88–.98)	.87	.89	.59	.97	8.09	.15	.93 (.89–.98)
Individual items															
Item 1	**–**	**1.00**	**.88**	**.64**	**1.00**	**8.33**	**0**	**.94 (.89–.98)**	**.87**	**.82**	**.46**	**.97**	**4.85**	**.16**	**.84 (.73–.96)**
Item 2	–	.72	.86	.54	.93	5.25	.32	.79 (.66–.92)	1.00	.87	.58	1.00	7.63	.00	.94 (.89–.98)

IPV = intimate partner violence; J-IPV = Jellinek Inventory for assessing Partner Violence; sens. = sensitivity; spec. = specificity; PPV = positive predictive value; NPV = negative predictive value; LR+ = positive likelihood ratio; LR− = negative likelihood ratio; AUC = area under the curve; ROC = receiver operator characteristics; CI = confidence interval.

### Effectiveness of the J-IPV for the Assessment of Severe IPV Victimization

Finally, psychometric properties of the J-IPV for detecting *severe* physical IPV victimization were determined (Table 5). In study 1, a positive answer to J-IPV item 1 led to both highest sensitivity (1.00) and highest NPV (1.00) in combination with largest AUC (.94), good specificity (.88) and acceptable PPV (.64). Furthermore, LR+ was 8.33 and LR− was.00. In study 2, these results were replicated; when item 1 was used as indicator for severe IPV victimization, the following results were found: sensitivity = .87; NPV = .97; AUC = .84; specificity = .82; PPV = .46; LR+ = 4.85; LR− = .16. However, optimal results were obtained when a positive answer to J-IPV item 2 was used as an indicator for severe IPV victimization (sensitivity = 1.00; NPV = 1.00; AUC = .94; specificity = .87; PPV = .58; LR+ = 7.63; LR− = .00). ROC-curves are again displayed in Charts S1.

## Discussion

The aim of the present study was to validate and cross-validate the J-IPV by determining its sensitivity, NPV, AUC, specificity, PPV, LR+, and LR−. It was decided that sensitivity and NPV, which we considered the most important properties of the J-IPV, should be at least ≥.80. Based on the results of the two studies, it is suggested to use the following cutoffs for the different purposes of the J-IPV. 1) To detect *any* IPV perpetration, we advise a cutoff of 1 since this yielded highest sensitivity (.80 and.84, respectively) and NPV (.85 and.82, respectively). In other words, 80/84% of IPV perpetrators screened positive for IPV perpetration on the J-IPV; after screening negative, 85/82% of the participants had indeed not committed IPV. 2) To detect *severe* IPV perpetration, optimal results were found for a cutoff of 2, which resulted in sensitivity and NPV of 1.00 in both studies. 3) To detect *any* IPV *victimization*, we recommend using a cutoff of 1, which resulted in highest sensitivity (.83 and.80) and NPV (.87 and.86). It is noticeable that the same cutoff was suggested for detecting any IPV perpetration; this could be explained by the fact that in about 70% of the cases IPV was reciprocal (see Table 3). 4) To detect *severe* IPV victimization, we recommend using a positive answer to J-IPV item 1 (study 1: sensitivity = 1.00; NPV = 1.00; study 2: sensitivity = .87; NPV = .97).

For the other psychometric properties, the following results were found for the different cutoffs. AUCs that varied between.84 and.94 indicated that there was an 84–94% chance that a randomly selected perpetrator/victim of any/severe IPV scored higher on the J-IPV than a randomly selected patient that did not perpetrate/experience any/severe IPV. Further, specificities ranged from.79–.88, indicating that 79–88% of factual non-IPV perpetrators screened indeed negative. PPV’s were found between.45–.80, indicating that 45–80% of all positive screening participants actually committed/experienced any/severe IPV in the past year. PPV’s of the J-IPV to detect severe IPV perpetration and victimization were on the low side (severe perpetration:.56 and.45; severe victimization:.64 and.58). However, we do not consider this problematic. The J-IPV is developed to screen for IPV and to identify patients in whom IPV should be further assessed. We argue that additional assessment of IPV in patients who are *not* factually involved in severe IPV is preferred above missing patients who are indeed involved in severe IPV. Moreover, patients who screened positive for severe IPV but were in fact not involved in *severe* IPV, were likely involved in non-severe IPV. Finally, likelihood ratios, ranging from 4.17–8.33 (LR+) and from 0–.25 (LR−), demonstrated that participants who screened positive were 4–8 times more likely than patients who screened negative to have perpetrated/experienced any/severe IPV, and that after screening negative, the odds were 0–25% to have perpetrated/experienced any/severe IPV. In sum, two independent studies demonstrated that the J-IPV is a valid screener with sensitivities and NPV’s ≥.80 to detect IPV in patients in substance abuse treatment. The second study replicated results from the first study, despite differences in population (relationship length and nationality), and despite the fact that the first study was conducted in a large city and the second in a smaller town. Although it was expected that the second study would perform less than the first study [48], this was hardly the case.

There were several limitations to the study. In the first place, both the J-IPV and the CTS2 rely on self-report of participants, which implies that the results do not automatically reflect the physical violence that actually took place. Participants may deny or minimize the violence they have committed or were victimized by. On the other hand, although people tend to underreport violence in their relationship when completing the CTS [22], research showed low correlations with social desirability [57]. Also, the purpose of the study was to investigate the J-IPV as an alternative for the CTS2 and *not* to study the predictive validity of the CTS2 and the J-IPV. Further, it is noticeable that more men than women participated in the two studies. However, men are overrepresented in substance abuse treatment and this does not reflect a selection bias. Finally, in the second study, 31 patients (19.1%) were excluded from the study because of logistic reasons, such as that the intaker had forgotten to administer the J-IPV or that there was not sufficient time in the intake to administer the J-IPV and CTS2.

In addition, there are several issues that should be addressed in future research. First, although the J-IPV demonstrated good psychometric properties in two different substance abuse treatment centers, these finding do not necessarily generalize to other settings (i.e., the J-IPV does not automatically possess external validity), since psychometric properties depend on the prevalence of a ‘disease’ in a population [46]. This emphasizes studying the validity of the J-IPV in other settings, such as forensic psychiatry, as well. Moreover, future research should include larger samples in order to narrow the 95% confidence limit of sensitivity and specificity. Using 98 participants, a sensitivity of.80 has a lower confidence limit of.65 [58]. Ideally, it should be increased up to.70 or.75 for which 204 and 756 participants are needed, respectively [58]. Another suggestion for future research is to obtain collateral data from participants’ partners on the CTS2 as well, but it should be taken into account that partner reports are not be reliable as well (e.g., [59]). Also, the order in which the J-IPV items are addressed would be interesting to study. Further, the J-IPV was developed to detect physical IPV. However, emotional and sexual IPV also have serious consequences for victims (e.g., [60], [61]). It would be of interest to study psychometric properties of the J-IPV to detect emotional IPV and to extend the J-IPV in order to detect sexual IPV. Also, it might be worth to investigate whether adding a third response category to the J-IPV (e.g., “sometimes”) may lower the threshold to admit IPV perpetration and/or victimization.

Since the J-IPV demonstrated good psychometric properties, it is recommended to routinely administer the J-IPV, which is available in the public domain, during intakes in substance abuse treatment facilities. If patients answer ‘yes’ to one or more of four J-IPV questions, IPV should be further assessed (stepped assessment), for instance, by administering the CTS2. Also, when using the J-IPV, various barriers on screening for IPV as reported by [29] are acknowledged. In the first place, recourse barriers (knowledge regarding screening and time constraints) are addressed: the J-IPV is very simple to use and score and administration takes only about 2 minutes. Moreover, since the J-IPV discriminates between any and severe IPV perpetration/victimization, it can be decided to assess IPV only in detail if patients screen positive for *severe* IPV perpetration and/or victimization if limited time or resources are available. Second, using the J-IPV would help overcome personal barriers (particularly personal discomfort) to screening for IPV; the J-IPV is conducted as structured interview, which makes it easier for intakers to address this sensitive topic. Finally, also patient-related barriers (the perception that patients with personality and/or psychosocial problems are difficult to screen) can be disputed on the basis of the present study. These problems are prevalent among patients in substance abuse treatment and no problems in administering the J-IPV have been encountered. An additional comment is that since [29] reported that other resource barriers (i.e., inadequate follow-up resources, lack of an office protocol, and inadequate locations and procedures for screening) were among the most cited barriers to screening for IPV, it is important that institutions have clear policies on screening for IPV and what to do with patients who screen positive for IPV.

After a positive J-IPV score and careful subsequent assessment, appropriate treatment for IPV perpetrators and/or victims should be arranged. However, to date, no evidence-based treatments addressing IPV perpetration exist [62], [63]. Nevertheless, research demonstrated that IPV perpetration decreases significantly after successful treatment for alcohol dependence [64], [65] and several researchers in the field argued that substance abusing IPV perpetrators should be allocated to a combined substance abuse - IPV treatment [66]–[69]. If it is decided to treat substance use disorders without directly addressing IPV, it is necessary to monitor IPV during the course of treatment, In addition, [66] demonstrated that combined substance abuse/partner violence treatment was effective in reducing IPV perpetration. For victims, help should be arranged by offering safety planning or providing advocacy interventions [70]. Yet another option is to refer patients to behavioral couples therapy, which has shown promising results for the treatment of IPV perpetration [62]. Although there are no evidence-based treatments addressing IPV perpetration, and although in some jurisdictions services for male IPV victims may be not be available, we argue that these are no legitimate reasons to abstain from screening for IPV. On the contrary, since it is known that IPV is prevalent in patients in substance abuse treatment, we consider it unethical *not* to screen for IPV in substance abuse treatment facilities.

## Supporting Information

Appendix S1. (DOCX)

Charts S1 Receiver operator characteristics (ROC)-curves to detect any and severe IPV perpetration and victimization for optimal scoring methods.
(DOCX)
