# Supplementary material for: Screening on Perpetration and Victimization of Intimate Partner Violence (IPV): Two Studies on the Validity of an IPV Screening Instrument in Patients in Substance Abuse Treatment
Source: PLoS One. 2013 May 16;8(5):e63681. doi: 10.1371/journal.pone.0063681 (PMC3656036; doi:10.1371/journal.pone.0063681)
Supplement: Appendix S1. — (DOCX) [file pone.0063681.s001.docx]

**Jellinek – Inventory for assessing Partner Violence (J-IPV)**

People with substance use problems are often known to experience tension in the relationship with their partner. Patients who enter treatment in our institution are relatively often a victim of domestic violence and / or have acted aggressively themselves in an argument with the partner. Therefore, we routinely pose the following questions.

| 1. | Has it occurred in the past year that the situation with your partner got so out of hand that your partner has threatened you, or that he/she threatened to harm you? | Yes / No |
| --- | --- | --- |
| 2. | Has it occurred in the past year that the situation with your partner got so out of hand and that your partner has been physically abusive towards you and for instance hit or kicked you? | Yes / No |
| 3. | Conversely, has it occurred in the past year that the situation with your partner got so out of hand that you acted in a threatening way to your partner, or threatened to hurt him/her? | Yes / No |
| 4. | And has it occurred in the past year that the situation with your partner got so out of hand that you became physically violent and, for example, slapped, hit or kicked your partner? | Yes / No |

**Scoring the J-IPV**

The following cutoffs should be used when scoring the J-IPV:

- Detection of any IPV perpetration: cutoff = 1 (at least 1 of 4 questions is answered positive)
- Detection of any IPV victimization: cutoff = 1 (at least 1 of 4 questions is answered positive)
- Detection of severe IPV perpetration: cutoff = 2 (at least 2 of 4 questions are answered positive)
- Detection of severe IPV victimization: a positive answer to item 1

**The J-IPV is available in the public domain.**
