# Supplementary material for: Screening on Perpetration and Victimization of Intimate Partner Violence (IPV): Two Studies on the Validity of an IPV Screening Instrument in Patients in Substance Abuse Treatment
Source: PLoS One. 2013 May 16;8(5):e63681. doi: 10.1371/journal.pone.0063681 (PMC3656036; doi:10.1371/journal.pone.0063681)

1. Receiver operator characteristics (ROC)-curve to detect any IPV perpetration (study 1) for a cutoff of 1 on the Jellinek Inventory for assessing Partner Violence (J-IPV).


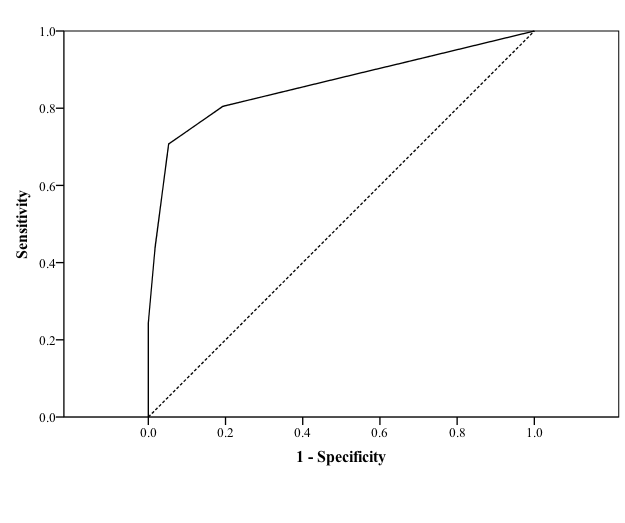


2. Receiver operator characteristics (ROC)-curve to detect any IPV perpetration (study 2) for a cutoff of 1 on the Jellinek Inventory for assessing Partner Violence (J-IPV).


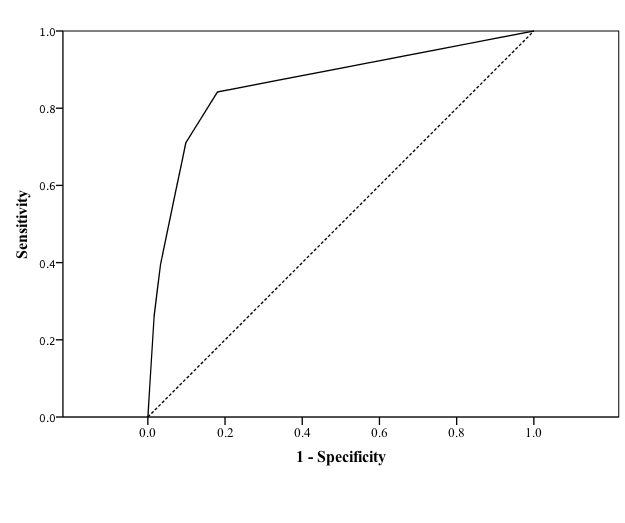
3. Receiver operator characteristics (ROC)-curve to detect severe IPV perpetration (study 1) for a cutoff of 2 on the Jellinek Inventory for assessing Partner Violence (J-IPV).


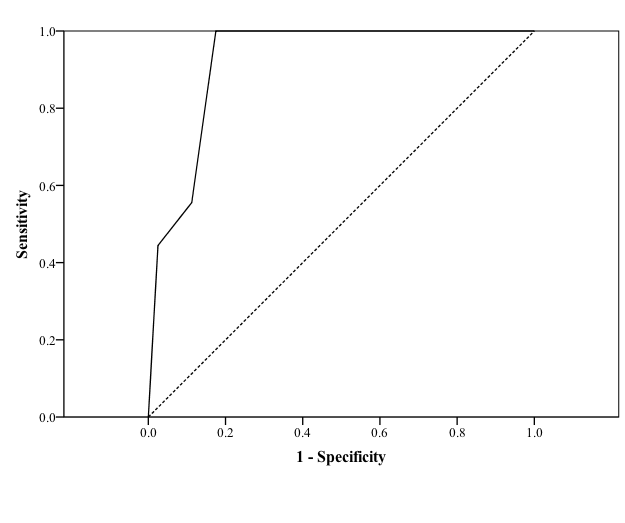


4. Receiver operator characteristics (ROC)-curve to detect severe IPV perpetration (study 2) for a cutoff of 2 on the Jellinek Inventory for assessing Partner Violence (J-IPV).


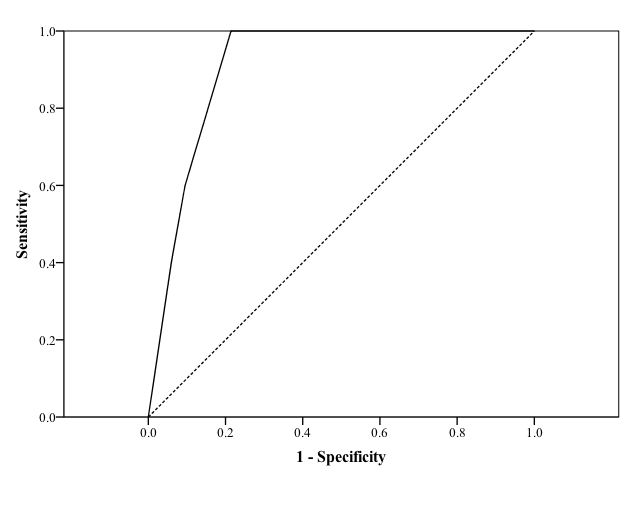
5. Receiver operator characteristics (ROC)-curve to detect any IPV victimization (study 1) for a cutoff of 1 on the Jellinek Inventory for assessing Partner Violence (J-IPV).


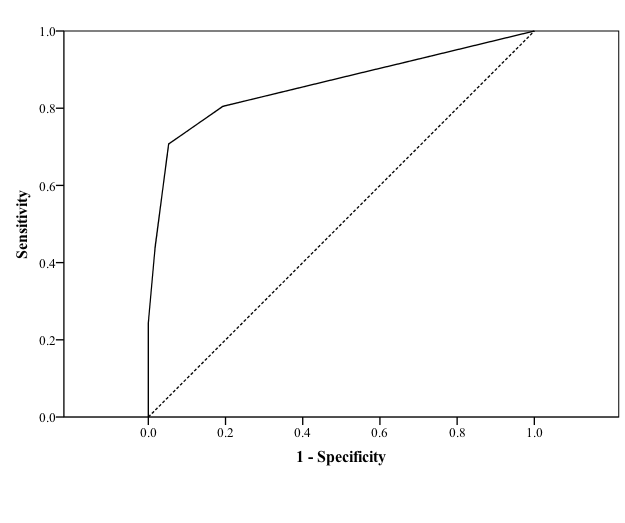


6. Receiver operator characteristics (ROC)-curve to detect any IPV victimization (study 2) for a cutoff of 1 on the Jellinek Inventory for assessing Partner Violence (J-IPV).


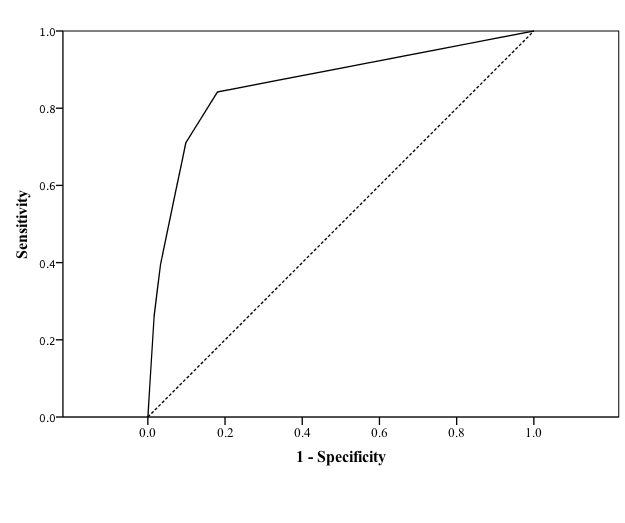


7. Receiver operator characteristics (ROC)-curve to detect severe IPV victimization (study 1) for a positive answer to item 1 of the Jellinek Inventory for assessing Partner Violence (J-IPV).


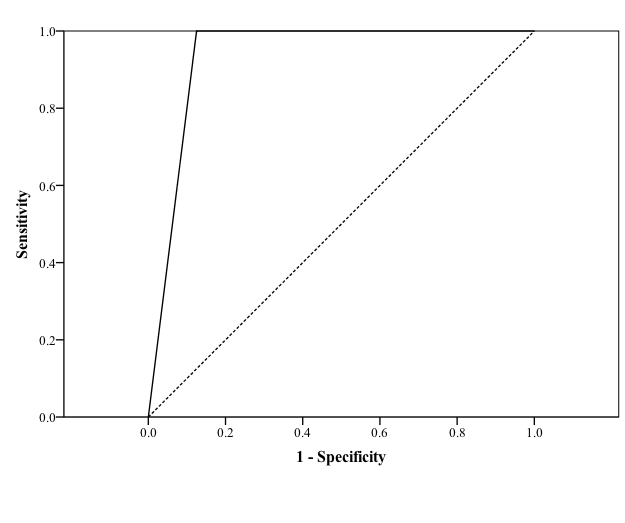


8. Receiver operator characteristics (ROC)-curve to detect severe IPV victimization (study 1) for a positive answer to item 1 of the Jellinek Inventory for assessing Partner Violence (J-IPV).


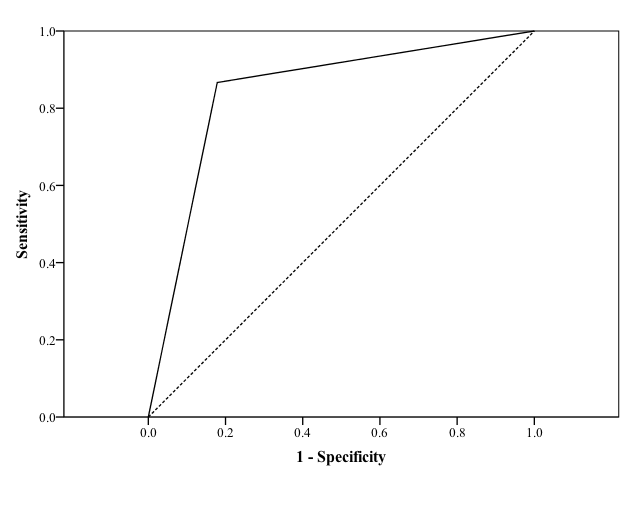

Supplement: Charts S1 — Receiver operator characteristics (ROC)-curves to detect any and severe IPV perpetration and victimization for optimal scoring methods. (DOCX) [file pone.0063681.s002.docx]
